# Release of leachable products from resinous compounds in the saliva of children with anterior open bite treated with spur

**DOI:** 10.1590/1678-7757-2022-0227

**Published:** 2023-02-03

**Authors:** Gabriel Antônio dos Anjos TOU, José Messias GOMES, Luiza Santana de Oliveira RINCO, Mônica YAMAUTI, Ivana Márcia Alves DINIZ, Fabiane PIRES, Marcella Emilia Petra SCHMIDT, Helvécio Costa MENEZES, Zenilda de Lourdes CARDEAL, Carla Beatriz Grespan BOTTOLI, Soraia MACARI

**Affiliations:** 1 Universidade Federal de Minas Gerais Faculdade de Odontologia Belo Horizonte Departamento de Odontologia Restauradora Minas Gerais Brasil Universidade Federal de Minas Gerais, Faculdade de Odontologia Belo Horizonte, Departamento de Odontologia Restauradora, Minas Gerais, Brasil.; 2 Universidade Federal de Minas Gerais Instituto de Ciências Exatas Departamento de Química Belo Horizonte Minas Gerais Brasil Universidade Federal de Minas Gerais, Instituto de Ciências Exatas, Departamento de Química, Belo Horizonte, Minas Gerais, Brasil.; 3 Hokkaido University School of Dentistry Department of Restorative Dentistry Sapporo Japan Hokkaido University, School of Dentistry, Department of Restorative Dentistry, Sapporo, Japan.; 4 Universidade Estadual de Campinas Instituto de Química Campinas São Paulo Brasil Universidade Estadual de Campinas, Instituto de Química, Campinas, São Paulo, Brasil.

**Keywords:** Methacrylates, Bisphenol-A glycidyl methacrylate, Dibutyl phthalate, Keratinocytes, Open bite

## Abstract

To evaluate the release of bisphenol-A glycidyl methacrylate (BisGMA), triethylene glycol dimethacrylate (TEGDMA), bisphenol A (BPA), and phthalates of the composite resin used in the bonding of spurs applied in the treatment of children with anterior open bite and its effects on human keratinocytes.

**Methodology:**

Saliva samples of 22 children were collected before spur attachment (baseline) and 30 minutes (min) and 24 hours (h) after spur bonding. Analysis was performed using high-performance liquid chromatography (HPLC) coupled to tandem mass spectrometry (HPLC–MS/MS) and gas chromatography coupled to mass spectrometry (GC–MS). Standardized resin increments were added to three different dilutions of the cell culture medium. Keratinocytes (HaCaT) were cultivated in the conditioned media and evaluated for cell viability (MTT) and cell scratch assay.

**Results:**

The levels of BisGMA (1.74±0.27 μg/mL), TEGDMA (2.29±0.36 μg/mL), and BPA (3.264±0.88 μg/L) in the saliva after 30 min, in comparison to baseline (0±0 μg/mL, 0±0 μg/mL, and 1.15±0.21 μg/L, respectively), presented higher numbers. After 24 h, the levels of the monomers were similar to the baseline. Phthalates showed no significant difference among groups. HaCat cells showed increased viability and reduced cell migration over time after exposure to methacrylate-based resin composites.

**Conclusion:**

Resin composites, used to attach spurs in children with anterior open bite during orthodontic treatment, release monomers after polymerization and can influence the behavior of human keratinocytes, even at very low concentrations. Orthodontists should be aware of the risks of the resinous compounds release and preventive procedures should be held to reduce patient exposure.

## Introduction

Methacrylate-based resin composites have been commonly used in bonding accessories in orthodontic treatment.^1^ The base monomer of the organic matrix is bisphenol-A glycidyl methacrylate (BisGMA), which, due to its high viscosity, is mixed with other dimethacrylates, such as triethylene glycol dimethacrylate (TEGDMA) and/or other monomers^2^ and additives, such as phthalates.^3^ Dental materials are susceptible to degradation when applied clinically and, as a consequence, may leach into the oral environment.^2,4-8^ The release of composite resin monomers is potentially hazardous^8-11^ with systemic^11^ and local effects on the oral mucosa, gum, and dental pulp.^8,12^ One of the products resulting from the decomposition of BisGMA is bisphenol-A (BPA), which is considered a xenoestrogen and can simulate the function of estrogen.^13^ In addition, exposure to BPA may lead to early sexual maturation in children,^14^ infertility^14^ increased risk of breast and prostate cancer,^15^ and changes in immune functions.^16^

Another leachable product found in dental materials is the phthalate,^3^ which is synthetic chemical ester of phthalic acid applied in the production of plastic materials.^17^ Exposure to phthalates is a risk because they are endocrine disruptors and can cause sexual changes in children.^17^

Studies demonstrated the release of resinous compounds in human saliva, urine, and blood after their use in restorative procedures, pit-and-fissure sealants, and orthodontics.^5,10,18,19^ Despite being similar, the chemical composition and clinical applications of these compounds directly influence the amount of leachable products release.^18^ Furthermore, in most restorative treatments performed on children, methacrylate-based materials are not indicated and are replaced by glass ionomer restorations.^20^ However, resin composites are still the first choice for bonding orthodontic accessories, even in children. Considering the great range of side effects of leachable products, more studies should be carried out in juveniles. At the moment, no previous studies quantified the amount of methacrylate, BPA, and phthalate release after bonding spurs with an orthodontic adhesive system in children at different experimental periods. This study aimed to analyze the release of BisGMA, TEGDMA, BPA, and phthalates such as diethyl phthalate (DEP), dibutyl phthalate (DBP), dibutyl phthalate (DiBP), dimethyl phthalate (DMP), and bis(2-Ethylhexyl) phthalate (DEHP), at different time points in the saliva of children after bonding spurs were used to treat anterior open bite and to simulate and analyze their effects on human keratinocytes (HaCaT) in cell cultures.

## Methodology

### Participants

**Figure 1:**
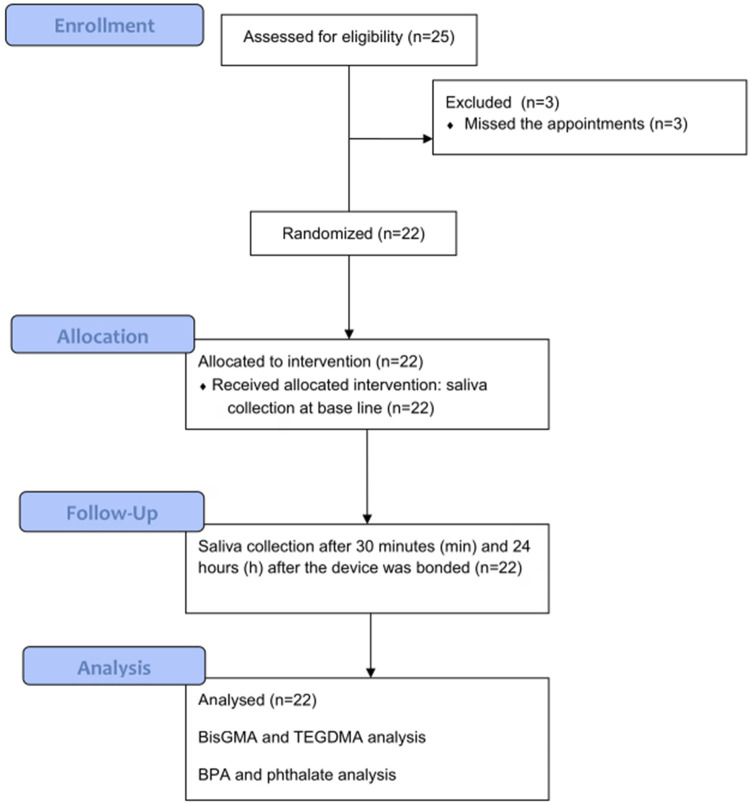
Participant flow diagram

For longitudinal analysis of the resinous compound leach quantification, patients systemically healthy with indications for interceptive orthodontic treatment and who had an anterior open bite were selected. The exclusion criteria was severe systemic alterations, use of antibiotics and anti-inflammatory drugs in the last three months, no oral malocclusion, and individuals who underwent restorations or sealants in the last 12 months. Sample size calculation was performed and a total of 25 participants were selected for the study. Three participants missed the appointments and the study remained with 22 participants that agreed to participate, including eight males and 14 females, with a mean age of 8.95±1.45 years (Figure 1). The consent forms were collected and signed by the legal guardians and the participants signed the assent forms.

Saliva collection was performed before the device was placed (baseline) and within 30 minutes (min) and 24 hours (h) after the device was bonded, following Tou, et al.^21^ (2022). The same operator performed the saliva collection and the attachment of the devices.

### Spur attachment

Relative isolation was performed, phosphoric acid (Fusion-Duralink, Angelus, Brazil) was applied for 30 seconds on the enamel surface, the Transbond XT adhesive system kit (3M, Unitek, Monrovia, California, USA) was applied following the manufacturer recommendations, and the bonding of the spurs (Morelli, Sorocaba, SP, Brazil) was performed on the lingual surface of the lower incisor, applying pressure in the center of the tool so that the composite resin could leach out on the spur sides. These excesses were removed. Two 40-second photo activations were performed with an LED lamp (Bluephase N, Ivoclar Vivadent Inc., Amherst, NY, USA. 1000 mW/cm2), one by the lingual side and the other by incisal.

### Saliva collection

To collect unstimulated saliva, volunteers were advised to sit comfortably with their heads slightly tilted down, allowing saliva to accumulate in their mouth, and then collect it in a glass vial. The collected saliva of each participant was immediately stored on ice and then at −80°C until the moment of analysis. A standardized procedure was performed with all saliva samples collected in the morning to avoid the change in composition during the day.

### BisGMA and TEGDMA analysis

The elution assay was adapted from Michelsen, et al.^22^ (2022) (Supplementary material). The Alliance 2695 liquid chromatography (Waters, Milford, MA, U.S.A) was used for the chromatographic analysis. The data acquisition and processing were performed using the Mass Lynx v. 4.1 software from Waters (Milford, MA, USA). For the identification and quantification, multiple reaction monitoring modes (MRM) were employed to confirm the presence of BisGMA and TEGDMA in the retention times (t_R_) using the *m/z *of the precursor ions and product ions. For BisGMA, the precursor ion (*m/z*) was 513.19 Da, the product ion (*m/z*) was 142.9 Da, cone voltage of 25.0 V, and collision energy of 20.0 eV. For TEGDMA, the precursor ion (*m/z*) was 286.97 Da, the product ion (*m/z*) was 112.9 Da, the cone voltage was 25.0 V, and the collision energy was 12.0 eV.

### BPA and phthalate analysis

The methodology applied for the analysis of BPA and phthalates (DEP, DBP, DiBP, DMP, and DEHP) was performed by gas chromatography coupled to a mass spectrometer (GC–MS) and was adapted from the method developed by Gomes, et al.^23^ (2020) (Supplementary material).

### *In vitro* experiment

To mimic the oral microenvironment in the condition of spur attachment, cell culture tests were performed to evaluate cell viability (3-[4,5-(dimethylthiazol-2-yl)-2,5-diphenyl tetrazolium bromide) (MTT assay)^10,24,25^ and cell migration (cell scratch experiment) of human keratinocytes (HaCaT)^24,25^ in the presence of products derived from orthodontic resin composites. A pilot study was performed to determine the amount of resin used for the *in vitro* experiment. The attachment of the four spurs was simulated and the amount of resin used for each appliance was weighted. The procedure was repeated three times and the mean value of 7.5 mg per spur was estimated. Four resin increments of 7.5 mg/each of the Transbond XT adhesive system kit (3M, Unitek, Monrovia, California, USA) were used to reproduce the amount of resin employed to attach the spur in the oral cavity of each child. The resin matrix was photoactivated two times for 40 seconds each with an LED lamp (1,000 mW/cm2, Bluephase N; Ivoclar Vivadent Inc., Amherst, NY, USA).

The four photoactivated increments were immersed in cell culture medium with three different volumes and dilutions: 5.5 mL (Dilution 1 – D1), 11.0 mL (Dilution 2 – D2), and 22.0 mL (Dilution 3 – D3) of basal medium and incubated for 30 min at 37°C. The dilutions were performed based on the salivary flow of a child up to 12-year-old that is approximately 0.7366 mL/min^-1^.^23^ Herein, the children’s average salivary volume in 30 min is represented by the greatest dilution tested (D3). Other two dilutions (D1 and D2) were tested (two and four times, respectively) as proof of concept of monomers cytotoxicity. The basal medium consisted of Dulbecco’s Modified Eagle Medium (DMEM) supplemented with 10% fetal bovine serum (FBS), penicillin-streptomycin (10,000 U/mL) (GIBCO, ThermoFisher Scientific, Grand Island, NY, USA).

For the MTT assay, human keratinocytes (HaCaT, Cell Line Service 300,493) were plated in quadruplicate at a cell density of 1×10^4^ cells/well in 96-well plates. Cell viability was performed following the manufacturer instructions at 24, 48, and 120 hours. For the cell scratch migration test, HaCaT cells were plated in triplicate at a density of 5×10^5^ cells/well in 6-well plates and were evaluated at 0, 24, and 48 h. At 0 h, a P200 tip wound was made in each well. Five images of each group were made, and the quantification of the percentage (%) of the wound area was measured using ImageJ software (National Institute of Health, Bethesda, MD, USA). In both experiments, a group of cells grown under ideal conditions treated exclusively with the basal medium was used as a control. The cells were cultured at 37°C in a humidified incubator with 5% CO_2_.

### Statistical analysis

The results are expressed as the mean ± standard deviation (S.D.). Data sets from the saliva and cell culture experiments presented a non-normal distribution (D’Agostino & Pearson normality test). For the BisGMA, TEGDMA, BPA, and phthalates, the differences among groups were analyzed by the paired Friedman nonparametric test followed by Dunn’s multiple tests. The differences among groups in the *in vitro* experiments were analyzed by the nonparametric Kruskal–Wallis test followed by Dunn’s multiple test. The value of P<0.05 was considered statistically significant.

## Results

### BisGMA and TEGDMA release 30 min after spur attachment

The levels of BisGMA (1.74±0.27 μg/mL) and TEGDMA (2.29±0.36 μg/mL) in the saliva increased after 30 min in comparison to baseline (BisGMA 0±0 μg/mL and TEGDMA 0±0 μg/mL) and 24 h groups (BisGMA 0±0 μg/mL and TEGDMA 0± 0μg/mL), without significant difference between the baseline and 24 h groups (Table 1).

**Table 1:** Analysis of levels of BisGMA and TEGDMA in the saliva of children using spur as a treatment to anterior open bite. A total of 22 children participated in this study. Friedman followed by Dunn’s Multiple Comparison Test. p<0.05 was considered statistically significant. Different letters mean the statistical difference among groups

	Baseline		30 min		24 h	
μg/mL	Mean	S.D. (±)	Mean	S.D. (±)	Mean	S.D. (±)
BisGMA	0.0^a^	0.0	1.747^b^	0.2776	0.0^a^	0.0
TEGDMA	0.0^a^	0.0	2.292^b^	0.3628	0.0^a^	0.0

### BPA leached after spur attachment, without significant differences in phthalate levels

The levels of BPA in the saliva of the children increased 30 min after spur attachment (3.26±0.88 μg/L) compared to baseline (1.15±0.21 μg/L) and 24 h after (0.77±0.11 μg/L), without significant difference between the baseline and 24 h groups (Table 2). We noted no significant differences in the levels of phthalates (DiBP and DBP) among the different time points (Table 2). The levels of DEP, DMP, and DEHP phthalates were not detected at any time point (data not shown).

**Table 2:** Analysis of levels of BPA, DiBP, and DBP in the saliva of children using spurs as a treatment for the anterior open bite. A total of 22 children participated in this study. No statistical difference in the amount of leached DiBP and DBP was detected during the periods of baseline, 30 minutes, and 24 hours after bonding the spur with the orthodontic adhesive. Friedman followed by Dunn´s Multiple Comparison Test. p<0.05 was considered statistically significant. Different letters mean the statistical difference among groups

	Baseline		30 min		24 h	
μg/mL	Mean	S.D. (±)	Mean	S.D. (±)	Mean	S.D. (±)
BPA	1.154^a^	0.2117	3.264^b^	0.8823	0.77^a^	0.1197
DiBP	17.56	2.977	23.75	4.172	14	2.436
DBP	8.316	1.061	8.901	1.197	6.333	0.9697

### Methacrylate-based leach of orthodontic resin reduced keratinocyte cell migration

**Figure 2:**
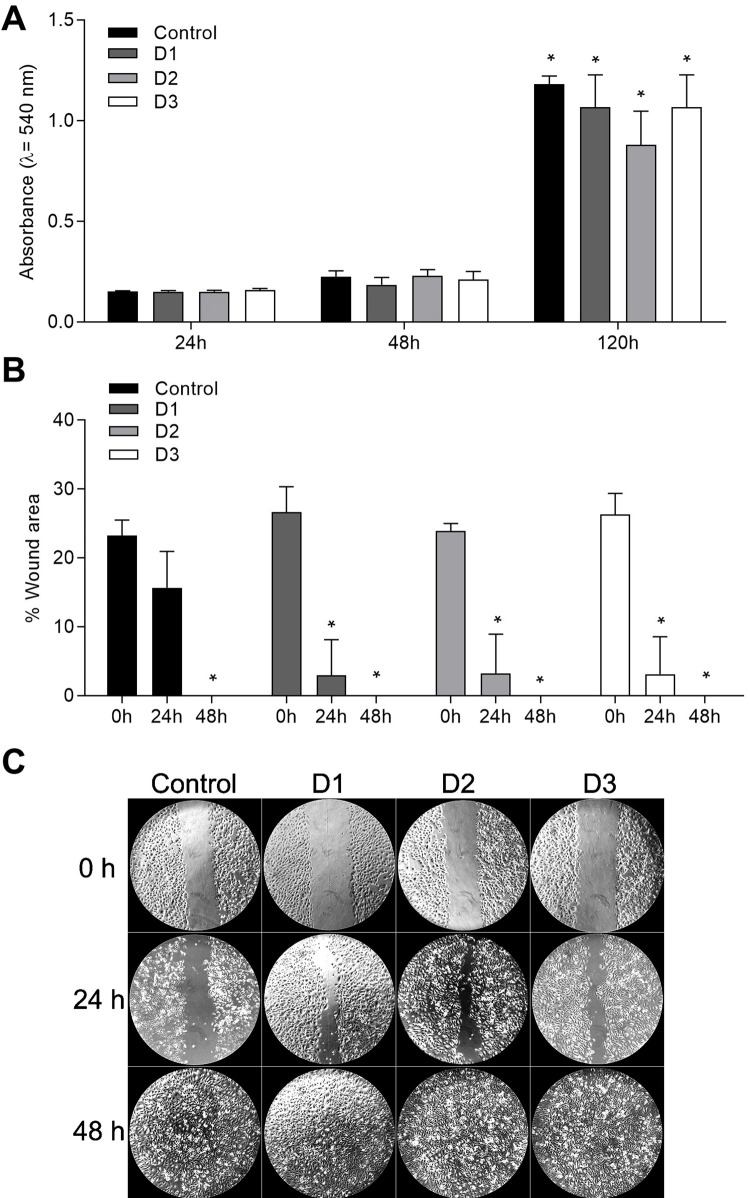
For the *in vitro* experiment, four photoactivated increments were immersed in a cell culture medium with three different volumes and dilutions: 5.5 mL (Dilution 1 – D1), 11.0 mL (Dilution 2 – D2), and 22.0 mL (Dilution 3 – D3). A group of cells grown under ideal conditions treated exclusively with the basal medium was used as a control group. A – Viability assay using Human keratinocytes (HaCaT) plated in quadruplicate for the MTT assay (3-(4,5-dimethylthiazol-2yl)-2,5-diphenyl tetrazolium bromide) for 24, 48, and 120 hours (h). B – Cell scratch migration test with HaCaT cells plated and evaluated at 0, 24, and 48 hours (h). C – Representative image of the cell migration analysis. Kruskal–Wallis nonparametric test followed by Dunn’s multiple tests. Only p<0.05 was considered statistically significant. A: The * means different results from four hours groups within the same treatment. B: The * means different results from 0-hour groups within the same treatment

The *in vitro* results showed that the viability significantly increased after 120 h compared to after 24 h. The 48 h group was similar to the 24 h and 120 h groups. We noted no difference among the dilutions at the same time point (Figure 2A). The percentage of the wound area was significantly decreased after 24 h compared to 0 h in the D1, D2, and D3 groups and recovered at 48 h in all experimental groups (Figure 2B and 2C).

## Discussion

The release of monomers and their byproducts from a resinous compound used in bonding orthodontic spurs was evaluated in the saliva of children with anterior open bite and *in vitro*. BisGMA, TEGDMA, and BPA, but not phthalates, were released into the oral environment 30 minutes after polymerization and the exposition to methacrylate-based resin-conditioned media increased the cell migration in human keratinocytes.

The presence of BisGMA, TEGDMA, BPA, and phthalates in the saliva of children over time was determined by HPLC and GC/MS. HPLC is the most widely accepted method for identifying and quantifying products from resinous dental materials because of its high efficiency in the evaluation of these compounds.^9,12,19^ However, the operational process and the techniques used for the extraction of sample compounds are widely variable,^9,18,23,26^ which may generate different findings among the studies. In addition, the amount and size of samples may vary in studies, including *in vitro* and *in vivo* analysis, so standardization of the samples may be challenging.^2,4,8,27^ The difference in the commercial brands used may also lead to differences in the number of components released by the materials and their genotoxic and cytotoxic potential.^4,10,19^ Our study was based on the experimental protocol of Moreira, et al.^18^ (2017) and Gomes, et al.^23^ (2020) to reduce the risk of error, thus increasing the capability of comparison among other studies.

Methacrylate-based dental materials are susceptible to degradation when applied clinically, so byproducts may be released into the oral environment.^8^ Compounds applied for bonding orthodontic accessories in the treatment of adults and pre-adolescent patients are among the degradable materials.^11,18^ We observed a significant pattern of increase in BisGMA, TEGDMA, and BPA concentrations after spur attachment in children, followed by a decrease that reached the initial values. An *in vitro* study quantified the elution of compounds from resin-based dental composites over one year and verified that BisGMA, HEMA, and UDMA were able to continuously elute from the materials up to 52 weeks after initial immersion.^28^ In accordance, other studies also identified component release within the first minutes after polymerization.^19^ Polydorou, et al.^4^ (2007) identified the presence *in vitro* of BisGMA and TEGDMA at 24 h and 7 days after polymerization.

The clinical application of each compound is also a factor that can interfere with the quantity of released byproducts.^5,8,11,29-32^ More fluid resinous materials present a higher percentage of leachable products released.^8,30^ Another important clinical factor to be considered is the volume of resin applied for different clinical situations and the time of the light curing process.^5^ The quantification of leachable products in most *in vivo* studies in the literature demonstrates that the release of byproducts is inversely proportional to the time after photoactivation of the resinous material^4,5,27,29,32^ and that most of the oligomer and monomer components are released within the first three to six hours after polymerization depending on the environment, with a rate of 80-100% release within the first 24 hours.^27^ Moreira, et al.^18^ (2017) identified significant BPA release in the saliva 30 minutes after light curing and did not find significant results at day seven, presenting much less BPA leach one month after the attachment of the bracket. Based on the literature and considering the small volume of resin required for bonding the spurs, in this study, a 7-day follow-up was performed, which was long enough to identify the leach of the monomers after the spurs attachment in the children’s saliva. Factors that interfere with the polymerization of resin materials, such as short light-curing time and distance between the material surface and light source, may contribute to a significant increase in the cytotoxic effects of the resin compounds.^26^ The polymerization of the adhesive system was standardized in both *in vivo* and *in vitro* experiments and performed by the same operator, thus avoiding experimental procedure bias.

Bisphenol A (BPA) is worldwide produced in large quantities and is a near-ubiquitous substance in today’s world. It is widely used for manufacturing polycarbonate plastics, which are found in the protective lining of plastic canned food items and plumbing pipes. BPA-based epoxy resins are also widely used for their adhesive properties in dental materials. BPA-detectable levels have been found in the urine of 93% of Americans aged six or older,^33^ with 0.014 μg/l being the maximum quantified BPA concentration in European found in potable water.^34^ Consequently, BPA has spread through our environment, making daily human exposure to BPA very intense. In this study, the baseline and 24 h after the spur attachment with resinous material groups presented BPA levels different from zero, with increased levels 30 minutes after bonding. These initial and 24 h after BPA values might be explained due to the patients’ environmental exposure to BPA from other possible sources. BPA exposure and average daily release were analyzed by previous studies in dental composite resins for dental restorations, glass ionomers, sealants, root canal sealers, and adhesives systems for orthodontic purposes.^5,8,11,29-32^ The average daily release of BPA from dental composite and resin glass ionomers ranged from 0.58±0.06 ng/g/day to 7.87±1.33 ng/g/day and 0 ng/g/day to 0.48±0.27 ng/g/day, respectively, in artificial saliva on the first day of an *in vitro* experiment, which is dependent on polymerization condition.^29^ In addition, BPA released from composite materials (one fissure sealant, two adhesives, and one root canal sealer) varied from 1.1±0.2 pg BPA/mg material to 21.4±2.3 pg BPA/mg material according to the type and amount of material and light-curing system used.^5^ Bagley, et al.^11^ (2021) demonstrated that the highest total BPA exposure was estimated for a dental restoration application compared to dental sealants and orthodontic adhesives, with the dental restoration application showing an average BPA exposure of 323.8 ng/treatment. TEGDMA may also arise from dental-cured products.^8,30,31^ TEGDMA light-cure dependent release from sealant samples ranged from 26.6 ppm to 84.98 ppm^31^ and TEGDMA *in-vitro *release from orthodontic adhesives was 31.7 µg/mL.^8,30^ Our results showed that 30 minutes after the spur bonding with the orthodontic adhesive system, the BPA levels increased almost three times compared to baseline levels and presented approximately 76% reduction after 24 hours.

Phthalates have been linked to health problems, such as early puberty in women,^35^ infertility, thyroid development, asthma, allergies, diabetes, increased risk to the breast and prostate, and changes in immune functions.^17^ This study evaluated the presence of phthalates in saliva; however, only DBP and DiBP were identified in the samples. The presence of phthalates in dental materials has already been identified.^36^ In our study, DEP and DMP were identified in concentrations below the limit of detection. The absence of these compounds in the saliva samples was not observed in other studies.^17,36^ A study found that DEP could be released from orthodontic resin aqueous media.^37^ In another study, DMP and DEP were detected in saliva samples of patients who had oral squamous cell carcinoma (OSCC) and in patients without this tumor.^23^ Therefore, the absence of DMP and DEP could be related to their degradation and absorption by saliva enzymes and oral tissues.^38^

Although the concentrations of DiBP and DBP can be related to the employment of resin, other sources can influence their concentration levels. The main sources of DiBP and DBP are not limited to food packages, cosmetics, and personal care products, but also solvents, plastic materials, and even dust particles that can be aspirated and ingested.^17,39^ The patients from this study did not have access to items that could be sources of phthalates in the sample collection. In our study, although not significant, DiBP and DBP were detected in all periods of salivary samples collected before and after the spur bonding. Similar events have been reported in other *in vitro* studies.^36^

The concern with the release of resin composite byproducts is that these components are toxic to tissues and cells.^10,25,40^ Cell culture studies demonstrated that BisGMA release simulated estrogen function in the body^13,41^ and that BPA was able to induce migration, proliferation, and estrogenic activity in MCF-7 breast cancer cells.^41,42^ TEGDMA exhibited excellent viscosity and copolymerization behavior and revealed considerable cell cytotoxic potency.^40^ We identified changes in cellular behavior in HaCat cells, showing an increase in cell migration in cultures exposed to methacrylates.

The resin-based products may also be cytotoxic to human gingival fibroblasts and keratinocytes and might interfere with cell proliferation and migration.^10,25,40,43^ The cytotoxicity of monomer release may occur in a dose-dependent manner and is dependent on the followability of the resin.^44,45^ Theilig, et al.^25^ (2000) evaluated the effect of BisGMA and TEGDMA on the induction of cell migration and proliferation of human fibroblasts and keratinocytes. It was identified that the presence of BisGMA without TEGDMA was able to significantly induce cell migration.^25^ In agreement with previous studies, the monomers present in the cell culture medium with the different dilutions increased the cell migration rate when compared to the control group.

A limitation of our study is that most of the cell culture materials consisted of plastic components and the levels of BisGMA, TEGDMA, and BPA were not measured in the cell culture medium at the different experimental time points. Another factor to be considered is the limitation of the *in vitro *experiment to represent the oral environment. During the *in vivo* experiment, the constant flow of saliva decreases the contact of human cells with the leached monomers, so the effects of these monomers on keratinocytes are expected to be lower than *in vitro*. However, through the *in vitro* analysis, the monomers are kept in constant contact with the cells in the medium, thus the toxicity of leachable compounds may increase in cell culture keratinocytes.

Issa, et al.^43^ (2004) evaluated the cell viability and cytotoxicity of resinous materials applied directly to the culture in human gingival fibroblasts by MTT and found that all monomers used in the manufacture of these materials, including BisGMA and TEGDMA, showed significantly reduced cell activity. Considering the immediate risk of monomers release from the adhesive system used to attach the spurs is related to epithelial cells, we tested cytotoxicity on HaCat monolayers. Under the conditions of the *in vitro* experiment, our results exhibited no significant difference in cell viability related to the monomer dilutions. On the contrary, we observed increased migration in the monomer-treated dilutions. Our results are aligned with a previous work reporting a slight interference of metallic ions and residual monomers on *in vivo* exfoliated buccal mucosa cells.^46^ Although methodologically dissimilar, Toy, et al.^46^ (2014) showed an increased number of bi-nucleated buccal epithelial cells, representing some morphological evidence of composite treated with Transbond stimuli after six months of assessment.

Treatment with lingual spurs in children with the anterior open bite is a method of known effectiveness and is well accepted by children.^21^ Although the resin used for spur bonding and spur attachment remains in contact with saliva at the edge of the appliance, patients were still exposed to BisGMA, TEGDMA, and BPA release, which could cause harm, especially in children. Nevertheless, considering that the peak of monomers leach at 30 min after its polymerization and subsequent release is almost null after 24 h, the level seems to be within the existing regulations and recommendations considered by authorities of 50 mg per kilogram per day.^19,47^ The impact of cumulative or low-dose effects over a long period should not be underestimated and should be taken into consideration. Therefore, it is recommended that orthodontists should be aware of the risks and preventive procedures to reduce patient exposure.

## Conclusion

Resin composites, used to attach spurs in children with anterior open bites during orthodontic treatment, release leachable products after polymerization and can influence the behavior of human keratinocytes in cell culture, even at low concentrations. Considering the health risks of leachable products, the release of derivatives from identified composites warns the dangers of compound applications in orthodontic treatments. More studies should be carried out to better understand the effects of these products on human cells.

## References

[1] Malkiewicz K, Turlo J, Marciniuk-Kluska A, Grzech-Lesniak K, Gasior M, Kluska M (2015). Release of bisphenol A and its derivatives from orthodontic adhesive systems available on the European market as a potential health risk factor. Ann Agric Environ Med.

[2] Ferracane JL (2011). Resin composite - state of the art. Dent Mater.

[3] Engel SM, Patisaul HB, Brody C, Hauser R, Zota AR, Bennet DH (2021). Neurotoxicity of ortho-phthalates: recommendations for critical policy reforms to protect brain development in children. Am J Public Health.

[4] Polydorou O, Trittler R, Hellwig E, Kummerer K (2007). Elution of monomers from two conventional dental composite materials. Dent Mater.

[5] Nys S, Duca RC, Vervliet P, Covaci A, Boonen I, Elskens M (2022). Bisphenol A release from short-term degraded resin-based dental materials. J Dent.

[6] Bationo R, Rouamba A, Diarra A, Beugré-Kouassi ML, Beugré JB, Jordana F (2021). Cytotoxicity evaluation of dental and orthodontic light-cured composite resins. Clin Exp Dent Res.

[7] Hassan R, Aslam Khan MU, Abdullah AM, Abd Razak SI (2021). A Review on current trends of polymers in orthodontics: BPA-Free and smart materials. Polymers.

[8] Lopes-Rocha L, Ribeiro-Goncalves L, Henriques B, Özcan M, Tiritan ME, Souza JC (2021). An integrative review on the toxicity of Bisphenol A (BPA) released from resin composites used in dentistry. J Biomed Mater Res B Appl Biomater.

[9] Van Landuyt KL, Nawrot T, Geebelen B, Munck J, Snauwaert J, Yoshihara K (2011). How much do resin-based dental materials release? A meta-analytical approach. Dent Mater.

[10] Taubmann A, Willershausen I, Walter C, Al-Maawi S, Kaina B, Golz L (2021). Genotoxic and cytotoxic potential of methacrylate-based orthodontic adhesives. Clin Oral Investig.

[11] Bagley BD, Smith JN, Teeguarden JG (2021). Risk assessment of predicted serum concentrations of bisphenol A in children and adults following treatment with dental composite restoratives, dental sealants, or orthodontic adhesives using physiologically based pharmacokinetic modeling. Regul Toxicol Pharmacol.

[12] Putzeys E, Cokic SM, Chong H, Smet M, Vanoirbeek J, Godderis L (2017). Simultaneous analysis of bisphenol A based compounds and other monomers leaching from resin-based dental materials by UHPLC-MS/MS. J Sep Sci.

[13] Gao H, Yang BJ, Li N, Feng LM, Shi XY, Zhao WH (2015). Bisphenol A and hormone-associated cancers: current progress and perspectives. Medicine.

[14] Rochester JR (2013). Bisphenol A and human health: a review of the literature. Reprod Toxicol.

[15] Maffini MV, Rubin BS, Sonnenschein C, Soto AM (2006). Endocrine disruptors and reproductive health: the case of bisphenol-A. Mol Cell Endocrinol.

[16] Sawai C, Anderson K, Walser-Kuntz D (2003). Effect of Bisphenol A on murine immune function: modulation of interferon-gamma, IgG2a, and disease symptoms in NZB X NZW F1 mice. Environ Health Perspect.

[17] Wang Y, Qian H (2021). Phthalates and their impacts on human health. Healthcare.

[18] Moreira MR, Matos LG, Souza ID, Brigante TA, Queiroz ME, Romano FL (2017). Bisphenol A release from orthodontic adhesives measured in vitro and in vivo with gas chromatography. Am J Orthod Dentofac Orthop.

[19] Kux BJ, Bacigalupo LM, Scriba A, Emmrich M, Jost-Brinkmann PG (2022). Elution study of acrylic monomers from orthodontic materials using high performance liquid chromatography (HPLC). J Orofac Orthop.

[20] Donly KJ, Garcia-Godoy F (2015). The use of resin-based composite in children: an update. Pediatr Dent.

[21] Tou G, Diniz IM, Ferreira MV, Mesquita RA, Yamauti M, Silva TA (2022). Evaluation of periodontal parameters and gingival crevicular fluid cytokines in children with anterior open bite receiving passive orthodontic treatment with a spur. Korean J Orthod.

[22] Michelsen VB, Kopperud HB, Lygre GB, Bjorkman L, Jensen E, Kleven IS (2012). Detection and quantification of monomers in unstimulated whole saliva after treatment with resin-based composite fillings in vivo. Eur J Oral Sci.

[23] Gomes JM, Almeida TF, Silva TA, Lourdes Cardeal Z, Menezes HC (2020). Saliva biomonitoring using LPME-GC/MS method to assess dentistry exposure to plasticizers. Anal Bioanal Chem.

[24] Oliveira RF, Marquiore LF, Gomes CB, Abreu PT, Ferreira LA, Diniz LA (2022). Interplay between epithelial and mesenchymal cells unveils essential proinflammatory and pro-resolutive mediators modulated by photobiomodulation therapy at 660 nm. Wound Repair Regen.

[25] Theilig C, Tegtmeier Y, Leyhausen G, Geurtsen W (2000). Effects of BisGMA and TEGDMA on proliferation, migration, and tenascin expression of human fibroblasts and keratinocytes. J Biomed Mater Res.

[26] Sunitha C, Kailasam V, Padmanabhan S, Chitharanjan AB (2011). Bisphenol A release from an orthodontic adhesive and its correlation with the degree of conversion on varying light-curing tip distances. Am J Orthod Dentofac Orthop.

[27] Ferracane JL (1994). Elution of leachable components from composites. J Oral Rehabil.

[28] Putzeys E, Nys S, Cokic SM, Duca RC, Vanoirbeek J, Godderis L (2019). Long-term elution of monomers from resin-based dental composites. Dent Mater.

[29] Tichy A, Simkova M, Vrbova R, Roubickova A, Duskova M, Bradna P (2021). Bisphenol A release from dental composites and resin-modified glass ionomers under two polymerization conditions. Polymers.

[30] Pelourde C, Bationo R, Boileau MJ, Colat-Parros J, Jordana F (2018). Monomer release from orthodontic retentions: an in vitro study. Am J Orthod Dentofac Orthop.

[31] Fung EY, Ewoldsen NO, Germain HA, Marx DB, Miaw CL, Siew C (2000). Pharmacokinetics of Bisphenol A released from a dental sealant. J Am Dent Assoc.

[32] Paula AB, Toste D, Marinho A, Amaro I, Marto CM, Coelho A (2019). Once resin composites and dental sealants release Bisphenol-A, how might this affect our clinical management? a systematic review. Int J Environ Res Public Health.

[33] Calafat AM, Ye X, Wong LY, Reidy JA, Needham LL (2008). Exposure of the U.S. population to Bisphenol A and 4-tertiary-octylphenol: 2003-2004. Environ Health Perspect.

[34] Arnold SM, Clark KE, Staples CA, Klecka GM, Dimond SS, Caspers N (2013). Relevance of drinking water as a source of human exposure to bisphenol A. J Expo Sci Environ Epidemiol.

[35] Wolff MS, Teitelbaum SL, Pinney SM, Windham G, Liao L, Biro F (2010). Investigation of relationships between urinary biomarkers of phytoestrogens, phthalates, and phenols and pubertal stages in girls. Environ Health Perspect.

[36] Munksgaard EC (2004). Leaching of plasticizers from temporary denture soft lining materials. Eur J Oral Sci.

[37] Bationo R, Jordana F, Boileau MJ, Colat-Parros J (2016). Release of monomers from orthodontic adhesives. AJADO.

[38] Dominguez-Romero E, Scheringer M (2019). A review of phthalate pharmacokinetics in human and rat: what factors drive phthalate distribution and partitioning?. Drug Metab Rev.

[39] Wormuth M, Scheringer M, Vollenweider M, Hungerbuhler K (2006). What are the sources of exposure to eight frequently used phthalic acid esters in Europeans?. Risk Anal.

[40] Geurtsen W, Leyhausen G (2001). Chemical-biological interactions of the resin monomer triethyleneglycol-dimethacrylate (TEGDMA). J Dent Res.

[41] Guzel KG, Naziroglu M, Ceyhan D (2020). Bisphenol A-induced cell proliferation and mitochondrial oxidative stress are diminished via modulation of trpv1 channel in estrogen positive breast cancer cell by selenium treatment. Biol Trace Elem Res.

[42] Kim JY, Choi HG, Lee HM, Lee GA, Hwang KA, Choi KC (2017). Effects of bisphenol compounds on the growth and epithelial mesenchymal transition of MCF-7 CV human breast cancer cells. J Biomed Res.

[43] Issa Y, Watts DC, Brunton PA, Waters CM, Duxbury AJ (2004). Resin composite monomers alter MTT and LDH activity of human gingival fibroblasts in vitro. Dent Mater.

[44] Thonemann B, Schmalz G, Hiller KA, Schweikl H (2002). Responses of L929 mouse fibroblasts, primary and immortalized bovine dental papilla-derived cell lines to dental resin components. Dent Mater.

[45] Al-Hiyasat AS, Darmani H, Milhem MM (2005). Cytotoxicity evaluation of dental resin composites and their flowable derivatives. Clin Oral Investig.

[46] Toy E, Yuksel S, Ozturk F, Karatas OH, Yalcin M (2014). Evaluation of the genotoxicity and cytotoxicity in the buccal epithelial cells of patients undergoing orthodontic treatment with three light-cured bonding composites by using micronucleus testing. Korean J Orthod.

[47] European Food Safety Authority (2022). Bisphenol A.

